# Mean Tear-Film Lipid Layer Thickness and Video Display Terminal Time as Risk Factors for Abnormal Blinking in Children

**DOI:** 10.3389/fmed.2021.785901

**Published:** 2021-12-06

**Authors:** Hui Zhao, Shi-Nan Wu, Zhe Cheng, Dong Xiao, Hui-Ye Shu, Qian-Min Ge, Tian Tian, Yi Shao

**Affiliations:** ^1^Xinhua Hospital Affiliated to Shanghai Jiao Tong University School of Medicine, Shanghai, China; ^2^Department of Ophthalmology, The First Affiliated Hospital of Nanchang University, Jiangxi Province Ocular Disease Clinical Research Center, Nanchang, China; ^3^Department of Radiology, The First Affiliated Hospital of Nanchang University, Jiangxi Province Medical Imaging Research Institute, Nanchang, China

**Keywords:** children, abnormal blinking, video display terminal time, dry eye, tear film alteration

## Abstract

**Objective:** To explore the risk factors for abnormal blinking in children and the role of the tear-film lipid layer thickness (LLT) as a function of duration of video display terminal (VDT) use in children.

**Methods:** Children attending the Optometry Clinic of Xinhua Hospital affiliated with Shanghai Jiao Tong University were recruited for the study between June 2019 and June 2020. Time spent viewing a VDT (VDTt) over the previous 6 months was recorded. Incomplete blinking (IB) and blinking rate were measured over a 10 s period using the Lipiview^®^ interferometer (Tear Science, Morrisville, NC, USA), and participants were allocated into groups with normal blinking (NBG, blink rate < 20 blinks/min) and abnormal blinking (ABG, blink rate ≥ 20 blinks/min). *T*-test, chi-square test and Mann-Whitney *U*-test were used to compare the differences in tear film (TF) stability indexes and meibomian gland function indexes between the two groups. Binary logistic analysis was used to analyze the risk factors for abnormal blinking and protective factors related to children's use of VDT, and receiver operating characteristic (ROC) curve analysis was also conducted.

**Results:** A total of 167 children were included, with no statistically significant differences in age or sex between the two groups. According to the *t*-test, VDTt was significantly higher in ABG than NBG, while TF stability indices including tear break up time, LLT and the height of the tear meniscus, were significantly higher in NBG than ABG (*P* < 0.001). The results also showed better meibomian gland function in NBG than ABG (*P* < 0.05). Binary logistic analysis showed that VDTt is an important risk factor for abnormal blinking, and the average of LLT (AVG) was found to be an important protective factor for children using a VDT for long periods, with a cut-off value of 1.5 h and 57.5 nm, respectively. ROC curve analysis showed that the area under the curve value of VDTt and AVG was 0.833 and 0.969, respectively (*P* < 0.001).

**Conclusion:** In children, VDTt is an important risk factor for abnormal blinking, and the AVG is an important protective factor for children using VDT for long periods.

## Introduction

A blink is a coordinated movement of eyelids closing and opening in a natural state. The normal blink rate is 10–15 blinks/min, each of 0.3–0.4 s duration, with inter-blink interval of 3–4 s. When the blink rate or amplitude exceeds an upper limit, it is considered abnormal. Abnormal blink as a chief complaint in children presenting at the ophthalmology clinic is most commonly secondary to other ocular abnormalities. In addition, some scholars have pointed out that abnormal blinks are closely related to the development of meibomian gland dysfunction (MGD) (1). Therefore, clinical optimization of blinking can effectively improve the symptoms of MGD patients. To date, some studies have shown that video display terminal (VDT) use is an important factor in dry eye (2). China has one of the highest portable VDT utilization rates in the world, with 847 million smart phone users in 2019, an average usage time of 27.9 h per week, 4% of users being children under the age of 10 and 16.9% being 10–19 years old, indicating that about 177 million children making extensive use of smart phones and VDTs (3). Long periods of VDT use are associated with discomfort symptoms such as dry eyes, ocular foreign body sensation, burning sensation and visual fatigue (4, 5). The various eye diseases related to VDT use are summarized in Table 1 (6–12). Relevant studies have shown that dry eye patients often have ocular surface epithelial damage, abnormal blinking and other clinical symptoms. Therefore, children may be at risk of abnormal blinking and possibly MGD (13) after prolonged use of VDT. Fenga et al. reported that long-term VDT users accompanied by MGD were more likely to have symptoms of eye discomfort and go to ophthalmology (11), which was consistent with the results of other scholars' studies (14).

**Table 1:** A variety of eye diseases caused by VDT.

**References**	**Year**	**Corresponding population**	**Eye diseases**
Moon et al. (6)	2016	Children	Dry eye
Ranasinghe et al. (7)	2016	Computer office workers	Computer Vision Syndrome
Basso et al. (8)	2006	VDT workers	Myopia
Ozawa et al. (9)	2015	Office workers aged 20–40 years	Eye fatigue
Lee et al. (10)	2016	Myopic patients with open-angle glaucoma	Visual field progression of open-angle glaucoma
Fenga et al. (11)	2008	VDT workers	Meibomian gland dysfunction
Chen et al. (12)	2017	Long term VDT user	Paracentral acute middle maculopathy

*VDT, video display terminal*.

There are many other external factors that can cause abnormal blinking. It has been reported that the main factors determining blink frequency are stimuli external to the eye, such as the state of the tear film (TF) on the surface of the cornea and conjunctiva, the excitation state of relevant receptors and environmental factors (15). As the outermost layer between the eye and the external environment, the ocular lipid layer of the TF plays an important role in reducing tear evaporation, increasing the stability of the TF and promoting the formation of TF on the ocular surface. A series of studies have shown a significant correlation between dry eye symptoms and tear-film lipid layer thickness (LLT) (16). Previous studies have focused on dry eyes following VDT use in adults, but few reports on meibomian glands (MGs) have involved long-term use of VDT in children. In addition, the scale of children's use of VDTs in China is expanding, and the age at which children begin to use VDTs is increasingly early. Children are unable to accurately describe their subjective symptoms, so conditions such as MGD may be neglected in children with long-term use of VDT. Abnormal blinking is a sign which can be observed by the clinician and does not need to be reported by the child. However, there are no published reports of the correlation between abnormal blinking and the time spent using VDT (VDTt), and there is a lack of research on the relationship between LLT, dry eye and abnormal blinking. Through the analysis of the correlation among VDT, dry eye and abnormal blinking, to provide guidance for the prevention and control of abnormal blink and dry eye in advance. Therefore, the purpose of this study was to explore the correlation between blink abnormalities in children and VDTt, the functional state of the MGs and the TF stability represented by LLT.

## Methods

### Patients and Examination

A total of 167 children aged 15 years or less attending ophthalmology outpatient clinics at Xinhua Hospital Affiliated to Medical College of Shanghai Jiao Tong University between June 2019 and June 2020 were enrolled. The blink rate was measured using the Lipiview^®^ interferometer, and subjects with a blink rate of ≥20 blinks/min were allocated to an abnormal blinks group (ABG), while those with a blink rate of <20 blinks/min were allocated to a normal blinks group (NBG) (17). Some data results were shown as mean ± standard deviation.

Participants were eligible if they met the following criteria: (1) no active inflammation of the eye and no eye drops used within the preceding three months; (2) no history of wearing corneal contact lenses, no history of laser or other ocular surgical operations, no history of ocular trauma, ocular chemical injury or burns; (3) no upper respiratory tract infection within the preceding two weeks; (4) no use of drugs affecting tears such as atropine, neostigmine or artificial tears within the last six months.

Children older than 15 years or with any of the following were excluded: (1) other ocular diseases that may cause ocular surface abnormalities such as abnormal eyelid position, proptosis, or pterygium; (2) other systemic diseases such as hyperthyroidism that affect tear production; or (3) developmental abnormalities. The study was compliant with the tenets of the Declaration of Helsinki, was approved by the Xinhua Hospital ethics committee and was registered with the Chinese Clinical Trial Registry (trial registration number: ChiCTR2000038908 and the approved number: XHEC-D-2018-103). Prior to each subject's participation, a guardian signed a declaration of paper informed consent.

Eye examination and other procedures were conducted in the following order: (1) The child and/or guardian was asked about the child's duration of VDT use over the preceding six months; (2) Lipiview^®^ measurements (including LLT and blink rate) were recorded; (3) tear meniscus height (TH) was measured and MG morphology was assessed using the Oculus Keratography 5M; (4) an ocular surface slit lamp examination was carried out to assess conjunctival congestion and fluorescein BUT, the Marx line (ML) score, MG expression and Meibum score, and corneal fluorescein staining score. All the examinations were finished in one day by the same ophthalmologist in the same dark room.

### Measurement of Tear-Film Lipid Layer Thickness (LLT), Incomplete Blinks (IB) and Blink Rate

The LLT of the TF (minimum, maximum and average), number of incomplete blinks (IB) and the blink rate over a 10 s period were measured using a Lipiview^®^ interferometer and during this procedure the child was encouraged to blink naturally (18). The equipment can automatically calculate the number of blinks per min and the number of abnormal blinks per min.

### TH and MG Morphology by Oculus Keratography 5M

The TH was measured using the Oculus Keratograph 5M camera and related software (19). TH ≥ 0.2 mm indicates normal tear secretion, while TH < 0.2 mm indicates insufficient tear secretion. The upper and lower eyelids were inverted, the conjunctiva was fully exposed, and the images were recorded, analyzed and height calculated. MG dropout was scored using the same equipment according to the following scale: I (normal, with no MG deficiency); II (MG deficiency <1/3); III (MG deficiency 1/3–2/3); IV (MG deficiency > 2/3) (20).

### Slit Lamp Examination

To measure TF breakup time (BUT), 2 μL 1% sodium fluorescein preservative free solution was dropped into the lower conjunctival sac and the participant was instructed to blink several times for a few seconds. TBUT was measured three times, and the mean calculated.

Corneal fluorescence staining score (FL) was measured by considering the corneal surface as four quadrants: supra-nasal, infra-nasal, superior temporal, and inferior temporal, with each quadrant being scored I-IV for a total out of 12 points. Scoring criteria were: I (no staining; 0 point); II (mild scattered spot staining, 1–30 spots stained; 1 point); III (moderate staining, >30 spots stained but staining not fused; 2 points); IV (heavy staining or sheet staining of the entire cornea, spot staining fused, with filaments, or ulcers; 3 points) (21).

The Marx line (ML) score was calculated for the outer, middle, and inner thirds of the lower eyelid margin, and recorded as: I [entirely on the conjunctival side of the meibomian orifices (MOs)]; II (part of the ML touches the MOs); III (ML runs through all of the MOs); and IV (ML runs on the eyelid margin side of the MOs) (22–24).

To measure MG expression (MGE), the five glands at the center of the upper and lower eyelids were located and their orifices observed. Scores were based on the following scale: I (normal, meibum secretion from all glands with light pressure on the eyelid); II (secretion from 3 to 4 glands with light pressure on the eyelid; III (secretion from 1 to 2 glands with light pressure on the eyelid); IV: no secretion from any glands with light pressure on the eyelid) (25).

The quality of secreted meibum was scored (Meibum score) according to the following criteria: I (normal, clear, transparent lid ester); II (cloudy lid ester); III (cloudy lid ester with granules); IV (thick lid fat with a toothpaste-like consistency) (26).

### Statistical Analyses

Statistical analysis was conducted using SPSS (version 25.0) and R (version 4.0.5; package pROC version 1.18.0; package ggplot2 version 3.3.5) to carry out *t*-tests, Chi-square test and Mann-Whitney U test between groups. Binary logistic analysis was performed on the related factors according to the grouping. Receiver operating characteristic (ROC) curves and the area under the curve (AUC) were used to analyze sensitivity and specificity of risk factors as diagnostic indicators. *P* values of <0.05 were considered statistically significant.

## Results

### Patient Demographics

**Figure 1:**
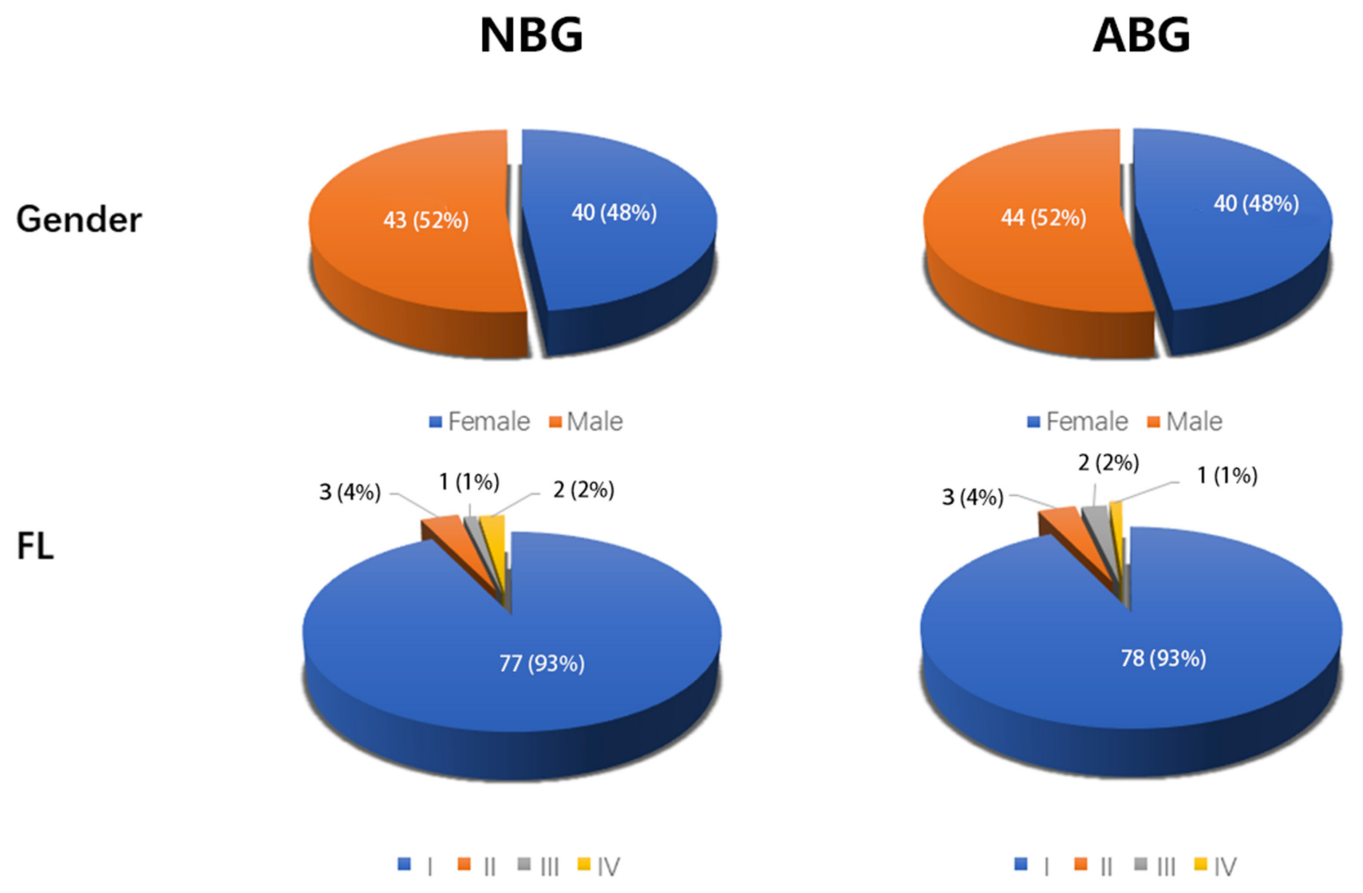
Clinical characteristics of NBG and ABG. *N* = 83 in NBG, *n* = 84 in ABG. NBG, normal blinking group; ABG, abnormal blinking group; FL, corneal fluorescence staining score.

A total of 167 children aged 15 years or younger were included in this case-control study. And 167 right eyes were examined in the study. The NBG included 40 females and 43 males (mean age 7.82 ± 2.66 years; range 2–15 years) and the ABG included 40 females and 44 males (mean age 8.14 ± 3.01 years; range 2–15 years). The two groups were statistically similar in age and gender (*P* = 0.462 and *P* = 0.941 respectively). The mean blink rate in the NBG was 14.60 ± 4.00, and this was significantly lower than the ABG blink rate of 30.00 ± 7.62 (*P* < 0.001). In addition, the frequency of incomplete blinking in ABG (23.71 ± 11.44) was significantly higher than that in NBG (13.95 ± 7.20; *P* < 0.001). The VDTt was also significantly different between the two groups, with longer times in ABG than in NBG (*P* < 0.001). Data are shown in Table 2; Figure 1.

**Table 2:** General information.

**Parameters**	**NBG**	**ABG**	***P* value^c^**
Age (years)^a^	7.82 ± 2.66	8.14 ± 3.01	0.462
Gender (F:M)^b^			0.941
Female	40 (48%)	40 (48%)	
Male	43 (52%)	44 (52%)	
Blinking rate^a^	14.60 ± 4.00	30.00 ± 7.62	<0.001
Incomplete blinking rate^a^	13.95 ± 7.20	23.71 ± 11.44	<0.001
VDTt (hours)^a^	1.03 ± 0.95	2.48 ± 1.08	<0.001

*P < 0.05 indicates statistical significance*.

a *Independent sample T test*.

b *Chi-squared test*.

c *Comparison between the NBG and ABG*.

*Data showed as mean ± standard deviation or n*.

*NBG, normal blinking group; ABG, abnormal blinking group; VDTt, video display terminal time*.

### Tear Film Stability

**Figure 2:**
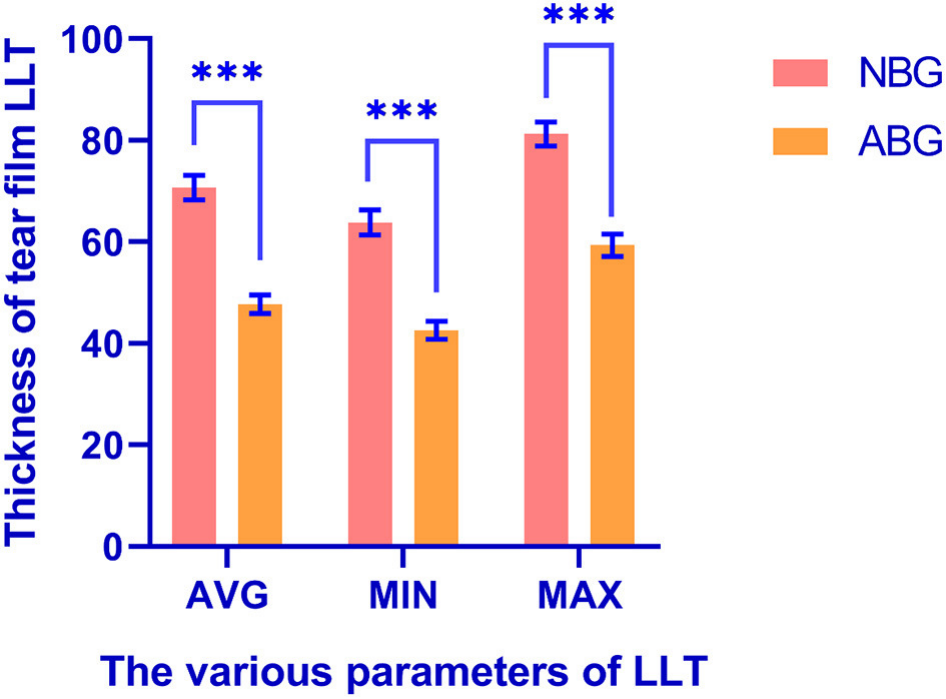
The difference of tear film LLT between NBG and ABG. ^***^There was a significant difference between the two groups. LLT, lipid layer thickness; NBG, normal blinking group; ABG, abnormal blinking group; AVG, the average of tear film lipid layer thickness; MIN, the minimum of tear film lipid layer thickness; MAX, the maximum of tear film lipid layer thickness.

The TH measure provided an indication of the quantity of TF. The TH and BUT of ABG was significantly lower than NBG, (TH 0.18 ± 0.07 and 0.21 ± 0.08; BUT 4.79 ± 2.65 and 6.25 ± 2.63; *P* < 0.001) (Table 3). LLT was assessed using mean and range. All LLT indices were significantly lower in ABG than NBG (mean 47.70 ± 16.88 and 70.70 ± 22.05; minimum 42.58 ± 16.40 and 63.81 ± 22.33; and maximum 59.35 ± 20.18 and 81.25 ± 21.27, respectively; all *P* < 0.001). No significant between-groups difference was found in FL (*Z* = −0.032, *P* = 0.974, Table 3) (Figure 2).

**Table 3:** The index of tear film.

**Parameters**	**NBG**	**ABG**	**Z^c^**	***P* value^c^**
FL^a^		−0.032	0.974	
I	77 (93%)	78 (93%)		
II	3 (4%)	3 (4%)		
III	1 (1%)	2 (2%)		
IV	2 (2%)	1 (1%)		
BUT(s)^b^	6.25 ± 2.63	4.79 ± 2.65	-	<0.001
TH (mm)^b^	0.21 ± 0.08	0.18 ± 0.07	-	<0.001
AVG^b^	70.70 ± 22.05	47.70 ± 16.88	-	<0.001
MIN^b^	63.81 ± 22.33	42.58 ± 16.40	-	<0.001
MAX^b^	81.25 ± 21.27	59.35 ± 20.18	-	<0.001

*P <0.05 indicates statistical significance*.

a *Mann-Whitney U test*.

b *Independent sample T test*.

c *Comparison between the NBG and ABG*.

*Data showed as mean ± standard deviation or n*.

*NBG, normal blinking group; ABG, abnormal blinking group; FL, corneal fluorescence staining score; BUT, tear film breakup time; TH, tear meniscus height; AVG, the average of tear film lipid layer thickness; MIN, the minimum of tear film lipid layer thickness; MAX, the maximum of tear film lipid layer thickness*.

### Meibomian Gland Function

**Figure 3:**
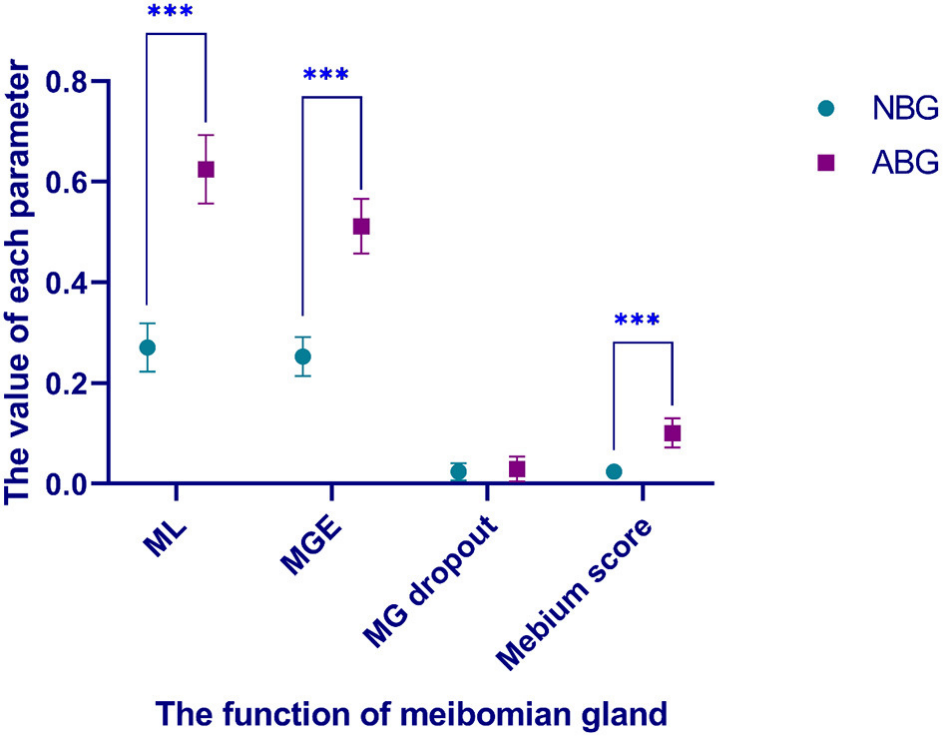
Comparison of meibomian gland function between NBG and ABG. ^***^There was a significant difference between the two groups. NBG, normal blinking group; ABG, abnormal blinking group; ML, marx line; MGE, meibomian gland expression; MG dropout, meibomian gland dropout degree.

MG dropout, meibum score, MGE and ML were used to evaluate the morphology and function of MG. Although there was no significant difference in MG dropout between the two groups (*Z* = −0.012, *P* = 0.990). ML (*Z* = −3.886, *P* < 0.001), MGE (*Z* = −3.798, *P* < 0.001) and Meibum scores (*Z* = −2.411, *P* = 0.016) were lower in the NBG than in the ABG. More detailed information is shown in Table 4; Figure 3.

**Table 4:** The function of meibomian gland.

**Parameters**	**NBG**	**ABG**	**Z^b^**	***P* value^b^**
ML^a^			−3.886	<0.001
I	59 (71%)	36 (43%)		
II	3 (4%)	5 (6%)		
III	21 (25%)	36 (43%)		
IV	0 (0%)	7 (8%)		
MGE^a^			−3.798	<0.001
I	51 (61%)	28 (33%)		
II	22 (27%)	34 (40%)		
III	10 (12%)	18 (21%)		
IV	0 (0%)	4 (5%)		
MG dropout^a^			−0.012	0.990
I	81 (98%)	82 (98%)		
II	0 (0%)	1 (1%)		
III	2 (2%)	0 (0%)		
IV	0 (0%)	1 (1%)		
Mebium score^a^			−2.411	0.016
I	80 (97%)	72 (86%)		
II	2 (2%)	7 (8%)		
III	1 (1%)	5 (6%)		

*P < 0.05 indicates statistical significance*.

a *Mann-Whitney U test*.

b *Comparison between the NBG and ABG*.

*NBG, normal blinking group; ABG, abnormal blinking group; ML, marx line; MGE, meibomian gland expression; MG dropout, meibomian gland dropout degree*.

### Binary Logistic and ROC Curve Analysis

**Figure 4:**
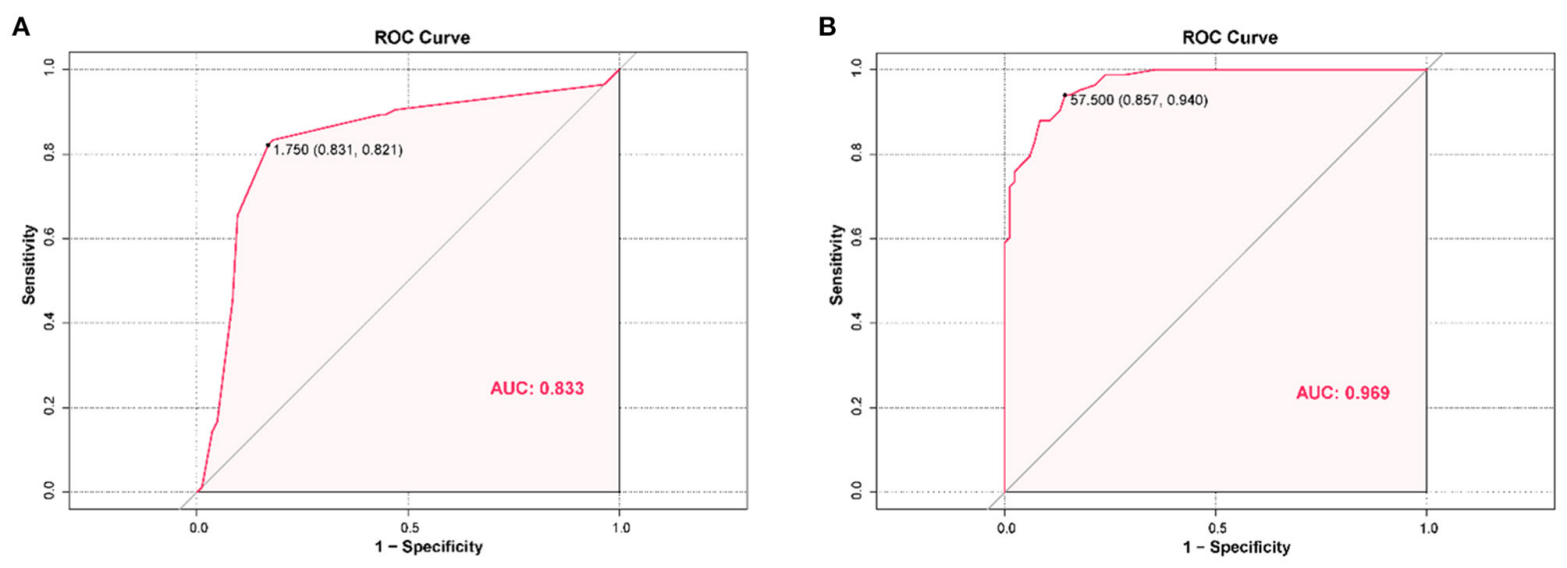
**(A)** The receiver operating characteristics (ROC) curves of risk factors for detecting children with abnormal blinking. ROC curves of VDTt showed that the AUC value was 0.833 (*P* < 0.001). The sensitivity and specificity were 82.7 and 82.5%, respectively. **(B)** The receiver operating characteristics (ROC) curves for factors associated with prolonged use of VDT in children. ROC curves of AVG showed that the AUC value was 0.969 (*P* < 0.001). The sensitivity and specificity were 85.7 and 94.0%, respectively. VDTt, video display terminal time; AVG, the average of tear film lipid layer thickness; AUC, area under the curve.

Binary logistic multivariate analysis based on ABG and NBG grouping showed that VDTt was an important risk factor for abnormal blinking. Based on an odds ratio of 3.486, it can be inferred that the risk of abnormal blinking after long-term use of VDT is 3.486 times higher than that of people who have not used VDT for a long time. ROC curve analysis was used to further explore this risk factor, and showed an AUC value for VDTt of 0.833. Based on the ROC curve, cut-off value of 1.5, sensitivity of 0.827, and specificity of 0.825 were determined (*P* < 0.001) (Tables 5, 6; Figure 4A).

**Table 5:** Binary logistic analysis results under different groups.

**Factor**	**Cut-Off value**	**Sensitivity (%)**	**Specificity (%)**	**AUC**	***P* value**
VDTt^a^	1.5	82.7	82.5	0.833	<0.001
AVG^b^	57.5	85.7	94.0	0.969	<0.001

a *The results are grouped according to a bound of 20 blinks per min*.

b *The results are grouped according to a daily bound value of 1.5 h of VDT usage*.

*P < 0.05 denoted statistical significance*.

*B, coefficient of regression; OR, odds ratio; CI, confidence interval; VDTt, video display terminal time; AVG, the average of tear film lipid layer thickness*.

**Table 6:** The Cut-off Value, sensitivity, specificity, and AUC of factors for the different grouping conditions.

**Factors**	**B**	**OR**	**OR (95% CI)**	***P* value**
VDTt^a^	1.249	3.486	2.410–5.042	<0.001
AVG^b^	−0.272	0.762	0.687–0.845	<0.001

a *The results are grouped according to a bound of 20 blinks per min*.

b *The results are grouped according to a daily bound value of 1.5 h of VDT usage*.

*P < 0.05 denoted statistical significance*.

*VDTt, video display terminal time; AVG, the average of tear film lipid layer thickness; AUC, area under the curve*.

### Binary Logistic Analysis Results and ROC Curve With 1.5 h as Boundary-Value Grouping

In order to exclude the collinearity effect of VDTt on other covariates, data were grouped based on the 1.5 h cut off value, and binary logistic multivariate analysis was conducted. Mean LLT was found to be an important protective factor with an odds ratio of 0.762. To evaluate the clinical significance of mean LLT in differential diagnosis, further ROC curve analysis was performed. The AUC value corresponding to mean LLT was 0.969, with cut-off value of 57.5, sensitivity and specificity of 0.857 and 0.940 respectively (*P* < 0.001) (Tables 5, 6; Figure 4B).

## Discussion

**Figure 5:**
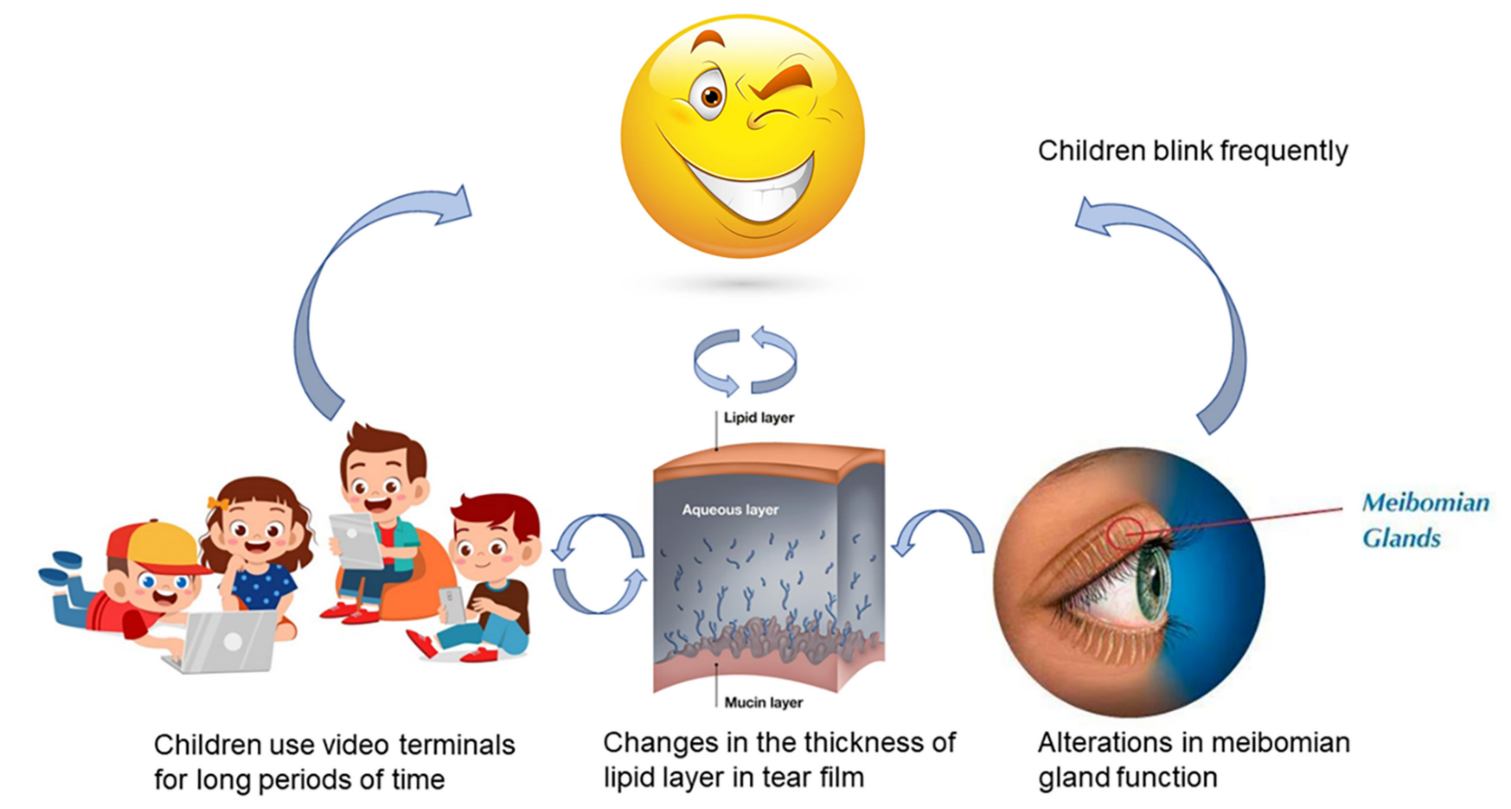
Frequent blinking in children was associated with prolonged use of terminal devices, thickness of tear film lipid layer, and alterations in meibomian gland function.

The present study identified the amount of time spent using a VDT as an important risk factor for abnormal blinking in children, and that VDTt of <1.5 h per day may be more conducive to children's eye health than longer periods. Moreover, statistical analysis pointed to mean LLT as an important protective factor for long periods of VDT use, with a bound value of 57.5 nm. This indicates that mean LLT ≤ 57.5 nm found in clinical ophthalmological examination would require consideration of the child's use of VDT and its possible impact on TF stability (Figure 5). According to the above clinical indicators, ophthalmologists could play a further important role in guiding children's eye health.

**Figure 6:**
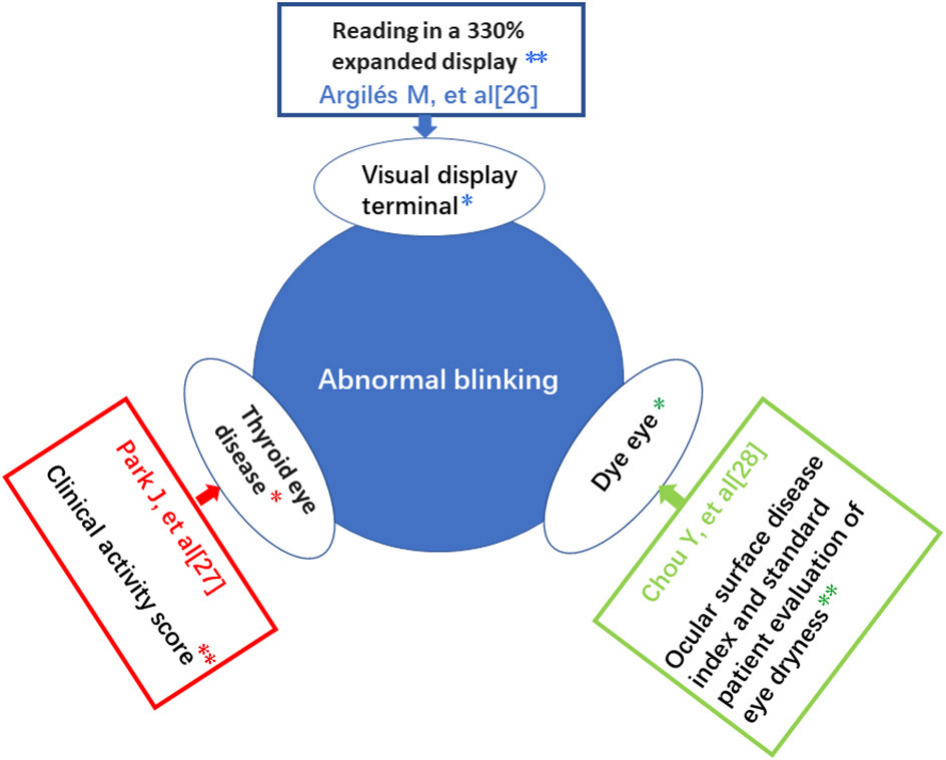
Summary of risk factors leading to abnormal blinking and their important influencing factors. ^*^Risk factors. ^**^Important influencing factors.

Previous research has shown that prolonged use of VDT could cause musculoskeletal pain, poor mental performance and psychological distress, and most commonly eye irritation (27). In China, a high risk factor for myopia in children is the use of VDT in close proximity, especially long periods of smartphone use (28). However, to date, little research exists on the correlation between abnormal blinking and the use of VDT. Since it is difficult for children to clearly express their symptoms, and children with xerophthalmia may have allergic conjunctivitis to varying degrees, these diseases may be misdiagnosed clinically (29). In a prospective study of frequent blinking in children, the most common cause was abnormality of the anterior segment or eyelid (30), but diverse causative factors exist and most are related to benign or self-limiting conditions (see Figure 6) (31–33). Careful medical history and clinical examination are important to determine the corresponding etiology and to provide timely and targeted treatment.

In this study, a significant risk factor for excessive blinking in children was prolonged video device use. Some scholars have pointed out that the prolonged use of VDT is an important cause of dry eyes, and found that warming moist chamber goggles have an excellent curative effect (34). There is a time-dependent oxidative stress response in ocular surface tissue, but the VDT durations in the present study are not sufficient to discern any damage to the ocular surface. Longer periods of VDT use may cause damage to the ocular surface. Perhaps short periods of use cause the decline of MG secretion function and the thinning of TF lipid layer, which leads to the deterioration of TF stability, but not the loss of MGs.

Numerous studies have shown reduced blink rates in long-term VDT users, as low as 7–11 blinks/min, with a significant increase in the percentage of incomplete blinks (35, 36). In children, with highly appealing entertainment and materials available to view on VDTs, blink rate is lower still, with the orbicularis oculi and Riolan muscles in children contracting completely around the eyelid gland opening. MG acini constantly synthesize and secrete meibum, which leads to the abnormal discharge of meibum, a decrease in MG secretion capacity and obstruction of the meibum (37, 38). This may be alleviated by the patient squeezing the eyes hard and increasing blink frequency. Previous research has shown that 60% of people who use VDT for long periods have significantly decreased blink rates, which in turn can lead to MGD (39). We hypothesize that children are more sensitive than adults to MG and TF abnormalities, with associated increased blink rate and abnormal blinking habits, and these signs can be misdiagnosed by clinicians as Tourette's syndrome.

Interestingly, studies have shown that incomplete blinking is associated with a two-fold increased risk of dry eye disease in patients with poor LLT (40). The TF lipid layer is composed of lipids secreted by meibomian glands, is located in the outermost layer of the TF, and has thickness in the range of 20–180 nm. As the lipid contact surface between the eye and the external environment, TF lipid layer can assist other TF components to inhibit tear evaporation, stabilize the air-tear surface between eye opening and blinks, and play a role in the first line of defense against bacterial invasion (41). The thickness of TF lipid layer is affected by a decrease of eyelid lipid or a change of tear composition.

In a recent study using Lipiview^®^, LLT was positively correlated with the number of normally secreting MGs, with LLT ≤ 60 nm in adults with dry eye, indicating a clinical application of MG assessment (33). This evaluation method has important clinical application value. In our study, BUT, TH and LLT were significantly lower in ABG than NBG. These finding suggest poor TF stability and a possibility of evaporative dry eye in children with abnormal blinking. For those using VDT for long periods, maintaining appropriate LLT could be a significant protective factor for ocular health. According to our statistical analysis, when the average LLT is reduced to 57.5 nm or less, TF stability may be significantly reduced in children using VDTs.

There are some limitations to our study. Firstly, abnormal blinking was assessed using the blink rate and number of incomplete blinks, and excessive blinking was not studied. Secondly, ocular surface morphology was observed over a short period, and longer observation time would help to compare different research results and support the research conclusion. Thirdly, the sample size of each group in the study was small, and larger studies would provide more certainty about the effect of VDTt on TF stability in children. Finally, the children involved in this study were all from the eastern Chinese city of Shanghai, so it is difficult to exclude the effect of air pollution on the ocular surface (42).

## Data Availability Statement

The original contributions presented in the study are included in the article/supplementary materials, further inquiries can be directed to the corresponding author/s.

## Ethics Statement

The studies involving human participants were reviewed and approved by Xinhua Hospital Affiliated to Medical College of Shanghai Jiao Tong University. Written informed consent to participate in this study was provided by the participants' legal guardian/next of kin.

## Author Contributions

HZ and S-NW were involved in the data curation and writing of the original draft. ZC, S-NW, and DX performed the data curation and formal analysis and participated in the writing and editing of the manuscript. H-YS was involved in the study conceptualization and methodology design. Q-MG and TT were involved in data validation and visualization. YS and HZ were involved in the study conceptualization, data curation, funding acquisition, and project administration. All authors have read and approved the final manuscript.

## Funding

This study was supported in part by the Medicine & Engineering Collaboration Research Fund of Shanghai Jiao Tong University (ZH2018QNB27). The funders have no role in the study design, data collection and analysis, decision on publishing, or preparation of the manuscript.

## Conflict of Interest

The authors declare that the research was conducted in the absence of any commercial or financial relationships that could be construed as a potential conflict of interest.

## Publisher's Note

All claims expressed in this article are solely those of the authors and do not necessarily represent those of their affiliated organizations, or those of the publisher, the editors and the reviewers. Any product that may be evaluated in this article, or claim that may be made by its manufacturer, is not guaranteed or endorsed by the publisher.
